# Identification of Gene Expression and Splicing QTLs in Porcine Muscle Associated with Meat Quality Traits

**DOI:** 10.3390/ani15091209

**Published:** 2025-04-24

**Authors:** Meng Zhou, Chenjin Ling, Hui Xiao, Zhiyan Zhang

**Affiliations:** National Key Laboratory for Swine Genetic Improvement and Germplasm Innovation Technology, Jiangxi Agricultural University, Nanchang 330045, China; lingchenjin@outlook.com (C.L.); xiaohui010709@foxmail.com (H.X.); bioducklily@hotmail.com (Z.Z.)

**Keywords:** pig, meat quality, eQTL, sQTL, *longissimus dorsi* muscle, RNA-seq, WGS, GWAS, colocalization

## Abstract

Understanding the genetic regulation of gene expression and splicing in muscle tissues is critical for elucidating the molecular mechanisms of meat quality traits. In this study, we integrated large-scale whole-genome sequencing and strand-specific RNA-seq data from 582 F2 hybrid pigs (White Duroc × Erhualian) to characterize the expression and splicing quantitative trait loci (eQTLs/sQTL) in *longissimus dorsi* muscle. We identified 11,058 cis-eQTL-associated genes (eGenes) and 5139 cis-sQTL-associated genes (sGenes), of which 29% of eGenes and 80% of sGenes were previously unreported in the PigGTEx database. Functional analyses revealed distinct genomic features: eQTLs were enriched near transcription start sites (TSSs) and associated with active TSS-proximal transcribed regions and enhancers, whereas sQTLs clustered at splice junctions, underscoring their distinct roles in gene expression and splicing regulation. Colocalization analysis of e/sQTLs with GWAS signals prioritized *PHKG1* as a key candidate gene (PPH4 > 0.9) for glycogen metabolism. Notably, we confirmed that an sQTL-driven alternative splicing event in exon 10 of *PHKG1* was significantly correlated with phenotypic variation (*R* = −0.39, *p* = 9.5 × 10^−21^). Collectively, this study provides novel insights into the genetic regulation of gene expression and alternative splicing in porcine muscle tissue, advancing our understanding of the molecular mechanisms underlying economically important meat quality traits.

## Simple Summary

In this study, we analyzed gene expression quantitative trait loci (eQTL) and splicing quantitative trait loci (sQTL) in the loin muscle of pigs. Utilizing genomic and transcriptomic data from 582 muscle samples, we identified thousands of loci associated with gene expression and splicing, many of which were previously unreported. Our results highlight distinct genomic architectures and regulatory mechanisms underlying eQTLs and sQTLs. Through colocalization analysis, we pinpointed the *PHKG1* (*Phosphorylase Kinase Catalytic Subunit Gamma 1*) gene as a strong candidate for influencing meat quality traits due to its role in glycogen metabolism. This work provides a valuable resource for deciphering genetic regulation in pig muscle and advancing strategies to improve meat quality traits.

## 1. Introduction

Meat quality is a crucial factor influencing both the meat processing industry and consumer preferences [1]. However, meat quality traits are complex quantitative traits regulated by intricate genetic networks [2], making genetic improvement a challenging yet essential goal in modern pig breeding. Despite genome-wide association studies (GWAS) have identified thousands of genetic variants linked to meat traits in pigs [3]. Most associated GWAS variants are located in noncoding regions and often reside within large linkage disequilibrium (LD) blocks, making it difficult to pinpoint the causal genes and variants [4,5]. This gap highlights the necessity of prioritizing functional genes and related variants, particularly in noncoding regions, as a central challenge in the post-GWAS era. Integrating molecular phenotypes, such as transcriptomic data, offers a promising strategy to address this limitation [6,7].

Expression and splicing quantitative trait loci (eQTLs/sQTLs) have emerged as powerful tools to bridge the gap between genotype and phenotype [6,8]. By linking genetic variants to gene expression and alternative splicing, these approaches enhance the biological interpretation of GWAS signals. Recent multi-species studies demonstrate that eQTL-GWAS integration substantially improves causal gene prioritization and explains a significant proportion of trait heritability [9,10,11]. In pigs, initiatives such as the porcine Genotype-Tissue Expression (PigGTEx) project have expanded multi-tissue eQTL/sQTL resources by aggregating publicly available RNA-seq datasets [12]. These efforts have linked over 80% of GWAS loci to candidate genes through molecular QTL analyses. However, inherent limitations persist, including heterogeneous genetic backgrounds [13], biases of variants calling from imputed RNA-seq genotypes [14], and—most critically—the blending of anatomically distinct muscle tissues in sample collections [15].

Tissue heterogeneity in porcine skeletal muscle is particularly significant. Muscle is composed of a diverse range of tissues distributed throughout the pig’s body, classified into over 47 distinct types [16], including the *semimembranosus* and *longissimus dorsi* muscles, each exhibiting unique physiological and molecular profiles [17,18]. Furthermore, the transcriptional divergence among various muscle types has been extensively documented [16,19], while the PigGTEx consortium aggregates diverse muscle tissues in its analyses, which may obscure region-specific regulatory mechanisms. Therefore, further research is essential to enhance the quality of eQTL/sQTL identification. Among the various muscle types, the *longissimus dorsi* muscle is distinguished by its superior palatability traits, including tenderness and intramuscular fat content, making it the most commercially valuable cut of pork [20]. Despite its economic significance, systematic investigations of its regulatory architecture through eQTL/sQTL analyses remain scarce, and its contribution to meat quality-associated GWAS signals remains poorly characterized [21,22,23].

To address these gaps, we constructed a high-resolution atlas of eQTL and sQTL in porcine *longissimus dorsi* muscle, uniformly sampled from the 6th to 7th rib of the back, using a cohort of 582 individuals from an F2 White Duroc × Erhualian cross-population. By integrating whole-genome sequencing and strand-specific RNA-seq data, we identified thousands of genetic variants influencing gene expression and splicing. We reveal novel eGenes and sGenes, many of which are absent in existing databases, and uncover divergent genomic features between eQTLs and sQTLs, reflecting their distinct roles in transcriptional regulation. Through colocalization analysis with GWAS signals for muscle glycogen-related traits, we prioritize candidate genes and demonstrate the functional relevance of alternative splicing in shaping phenotypic outcomes.

## 2. Materials and Methods

### 2.1. Animal Sampling and Sequencing

A total of 582 F2 individuals, comprising 351 boars and 231 sows, were collected in this study. The F2 population was developed following the previously described [24]. In summary, 17 Erhualian sows were mated with two White Duroc boars, and the final F2 population was established by crossing nine F1 boars with 59 F1 sows. All animals were born and raised at the experimental farm of the National Key Laboratory for Swine Genetic Improvement and Production Technology, Jiangxi Agricultural University (Nanchang, Jiangxi, China), under standardized housing and feeding conditions. Pigs were fed twice daily with a formulated diet containing 16% crude protein, 3100 kJ/kg digestible energy, 0.78% lysine, 0.6% calcium, and 0.5% phosphorus. Water was provided ad libitum via nipple drinkers. All F_2_ animals were transported to the commercial abattoir in Nanchang for slaughter. Prior to slaughter, pigs were fasted for 15–20 h with free access to water. Slaughter was performed at an average age of 240 ± 16 days, with body weights ranging from 67 to 131 kg.

Following slaughter, *longissimus dorsi* muscle samples were collected uniformly from the region between the 6th and 7th ribs on the left side of each animal. Samples were immediately frozen and stored at −80 °C until further analysis. All animals included in this study were healthy and had not received any antibiotic treatment within one month prior to sampling. All animal procedures were conducted in accordance with the National Standards of the People’s Republic of China and were approved by the Ethics Committee of Jiangxi Agricultural University (Approval No. JXAULL-2021-12-001).

Total DNA was extracted from the ear, blood, or muscle tissues using the phenolic chloroform method. DNA library construction and sequencing were performed using BGI platforms (Beijing Genomics institution, Shenzhen, China). Briefly, a minimum of 1 μg of extracted DNA was fragmented using ultrasound and Covaris technology (Life Technologies, Carlsbad, CA, USA). This was followed by A-tailing, adapter ligation, and size selection to enrich for insert sizes of 300–400 bp. After PCR amplification and cyclization, the sequencing library was constructed and sequenced on the DNBseq platform using PE150 mode. The raw sequencing data was filtered to remove the reads with low-quality bases and “N” sites using fastp (v0.23.0) [25] with default parameters.

Total RNA was extracted from the same 582 muscle samples using TRIzol (Invitrogen, Carlsbad, CA, USA). RNA quality metrics, including purity and integrity, were evaluated using a NanoDrop’m One spectrophotometer (Thermo Fisher Scientific, Waltham, MA, USA) and a Bioanalyzer 2100 system (Agilent Technologies, Santa Clara, CA, USA). Polyadenylated mRNA was enriched based on poly-T magnetic beads, followed by fragmentation and synthesis of strand-specific cDNA libraries. Library preparation included sequential steps of end repair, 3′-adenylation, adapter ligation, and size selection targeting 300–400 bp inserts. The amplified libraries were sequenced using the DNBseq platform in PE150 mode. Raw data was filtered out using quality control parameters consistent with the WGS process.

### 2.2. DNA Alignment and Variant Calling

The WGS reads were aligned to the Sscrofa11.1 [26] genome using the BWA MEM (v0.7.17-r1188) [27]. Duplicates generated from PCR amplification were removed using the Sambamba (v0.8.2) [28]. Subsequently, SNPs and indels (<50 bp) were identified using the GATK HaplotypeCaller (v4.1.4.1) following best practice recommendations [29] to generate a gvcf file for each sample. Then, all-sample gvcf files were joint called using the glnexus_cli (v1.4.3-0-gcecf42e) [30] to merge into a single population variant detection vcf file. Finally, we applied stringent filtering criteria for the identified variants, retaining only those that satisfied all of the following conditions: (i) minimum reads coverage depth 3×; (ii) Genotype quality score ≥ 10; (iii) biallelic variants; (iv) minor allele frequency (MAF) > 0.05 and missing genotype rates of <20%

### 2.3. Expression and Splicing Quantification

The RNA-seq reads were aligned to the Sscrofa11.1 genome using the HISAT2 (v2.2.0) [31] with a stand-specific option: --rna-strandness RF. Subsequently, we calculated raw read counts and normalized expression levels (TPM value) for 21,281 protein-coding genes based on the Sscrofa11.1 gene annotation (Ensemble v100) through featureCounts (v2.0.6) [32] (-s 2 to ensure strand specificity) and StringTie (v2.1.4) [33] (--fr for strand orientation), respectively. Genes with TPM > 0.1 and detectable expression in >20% of samples were retained for further analysis. TPM values were subsequently normalized using the Trimmed Mean of the M-value (TMM) method in edgeR [34], followed by an inverse normal transformation.

LeafCutter (v.0.2.9) [35] was employed to detect and quantify the local splicing events in protein-coding genes. Initially, junctions and intron clusters were extracted and defined across samples using the scripts “bam2junc.sh” and “leafcutter_cluster.py”, based on the HISAT2 alignments. For intron clustering, we required a minimum of 50 split reads per cluster and at least 0.1% of reads supporting any given junction within a cluster. The maximum allowed intron length was set to 500 kb, in accordance with LeafCutter’s default parameters. Subsequently, the “prepare_genotype_table.py” script was employed to gain intron excision ratios, filtering out introns present in <50% of individuals or with read counts < 58 (10% of the sample size). The intron excision ratios were then standardized and subjected to quantile normalization procedures, resulting in sample-wide percent spliced-in (PSI) values for downstream analyses.

### 2.4. Covariate Selection

To mitigate the influence of potential confounding factors on gene expression and intron excision ratios, we employed the probabilistic estimation of expression residual (PEER) method [36] to detect hidden confounders by utilizing the normalization gene expression and intron excision ratio matrices. Additionally, we used PLINK [37] with the parameters: --pca 30 --autosome to compute the top thirty principal components (PCs) of genotype data, ensuring that population genetic structure effects were accounted for using a genome-wide variation. Finally, based on their contributions, the top seven genotype PCs and the top five PEER factors were selected for cis-eQTL analysis, while the top ten PEER factors were chosen for cis-sQTL analysis (Supplementary Figure S1).

### 2.5. Estimating Cis-Heritability of Gene Expression and Intron Excision Ratio

Cis-heritability was estimated using 13,707 expressed genes and 66,208 intron clusters. Variants considered for gene expression heritability were those located within 1 Mb of the TSS of the corresponding genes, whereas those relevant to intron excision ratio heritability were defined as variants within 1 Mb of the associated intron clusters. The genetic relationship matrix (GRM) was constructed using GCTA (version 1.94.1) [38], incorporating these cis-variants linked to target genes or intron clusters. Heritability estimates were then obtained using the restricted maximum likelihood (REML) method with the -reml function in GCTA [38] while adjusting for the covariates mentioned above.

### 2.6. Cis-eQTL and Cis-sQTL Mapping

cis-eQTLs were identified using a linear model with TensorQTL (v1.0.5) [39] software, adjusting for the top five PEER factors and seven PCs. The cis-loci were restricted to variants within 1 Mb of the TSS of each expressed gene. By setting the option to “--mode cis_nominal” in TensorQTL (v1.0.5) [39], we evaluated all nominal significance levels for variant-to-gene association pairs. Additionally, a permutation mode was employed to calculate empirical *p*-values for gene expression using the “--mode cis” setting in TensorQTL (v1.0.5) [39]. After applying false discovery rate (FDR) correction [40] to beta-approximated empirical *p*-values, cis-eGenes were classified as those associated with at least one significant variant (FDR < 0.05).

The cis-sQTL mapping was conducted with TensorQTL (v1.0.5) [39], examining associations with variants situated within 1 Mb of intron clusters and their corresponding intron excision ratios. For this analysis, the top ten PEER factors and seven PCs were used as covariates. Unlike cis-eQTL mapping, a grouped permutation strategy was employed to jointly evaluate empirical significance thresholds across all intron clusters. For each gene, the most significant associated variant-intron cluster pair was designated as the primary cis-sQTL. After applying false discovery rate (FDR) correction [40] to beta-approximated empirical *p*-values, cis-sGenes were classified as genes containing any introns with at least one significant variant (FDR < 0.05).

To evaluate the accuracy of TensorQTL in detecting cis-eQTLs using linear models, we employed an independent linear mixed model (LMM)-based approach through fastGWA (GCTA v1.94.1) [39] to reanalyze cis-eQTLs and cis-sQTLs for muscle tissue. A sparse genetic relationship matrix (GRM) was constructed based on whole-genome variants. Similar to the TensorQTL analysis, the fastGWA implementation only considered variants located within ±1 Mb flanking regions of target genes for molecule QTL mapping when correcting for GRM and the corresponding covariates.

### 2.7. GWAS of Meat Quality Traits

The meat quality traits of 546 corresponding F2 individuals including total glycogen (TG) and residual glycogen (RG) were measured in *longissimus dorsi* muscles as described previously [41]. Whole-genome variants data and phenotype values were analyzed through linear mixed model-based GWAS implemented in GEMMA (v0.98.5) [42], with population stratification controlled by a genetic relationship matrix (GRM). LD-based variant pruning was performed using PLINK (v2.0) [37] with stringent parameters (-indep-pairwise 50 5 0.2) to ensure marker independence for GRM construction. The analytical framework incorporated fixed effects for sex and slaughter batches while adjusting for polygenic background GRM. The significance threshold for association was set to 5 × 10^−8^.

### 2.8. Colocalization and Quantitative Trait Transcripts (QTT) Analysis

To investigate potential co-localization between eQTL/sQTL variants and GWAS-associated loci related to meat quality traits in the F2 population, we used the GWAS summary statistics of total glycogen (TG) and residual glycogen (RG) for colocalization analysis. The co-localization assessment was conducted through the Bayesian framework-based Coloc package (v5.1.0) [43] where a posterior probability threshold (PPH4 > 0.80) for the co-localization hypothesis was established as the criterion for confirming shared genetic signals between transcriptional regulation variants (eQTL/sQTL) and glycogen-related trait association.

Similarly, we used an F7 hybrid population to validate the co-localization of the *PHKG1* gene sQTL with the RG GWAS signals. Briefly. A total of 301 F7 individuals from a heterogeneous pig population were generated by crossing eight founder breeds, including Bamaxiang (BX), Erhualian (EH), Laiwu (LA), Tibetan pig (TB), Duroc (DU), Landrace (LD), Large White (LW) and Pietrain pigs (PT). We designed a circular mating program to equally mix the ancestries of the eight founder breeds in individuals of the third and later generations as described previously [44,45]. All animals were housed in an environment similar to that of the F2 population, and the *longissimus dorsi* muscles were consistently obtained for WGS and RNA-seq sequencing. Using this data, we followed the above-mentioned consistency process for co-localization analysis.

To evaluate the correlations between *PHKG1* gene expression levels and intron excision ratios with six meat quality traits, we conducted a QTT analysis. The *PHKG1* gene expression levels and intron retention rates were adjusted for PCA and PEER factors, while phenotypic values were adjusted for sex, slaughter batch, and PCA. Statistical correlations and significance between the adjusted phenotypic values and molecular expression levels were analyzed using the lm function in R.

## 3. Results

### 3.1. Data Process and Quality

**Figure 1:**
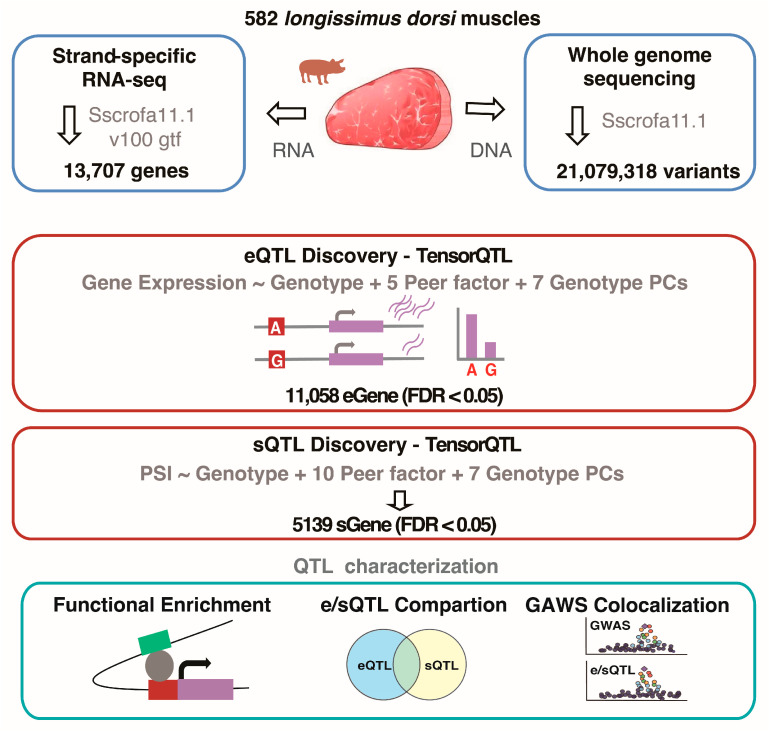
Overview of study design and analysis. Strand-specific RNA-seq and whole-genome sequencing were performed on 582 *longissimus dorsi* muscle samples for eQTL and sQTL mapping. eQTL and sQTL were independently identified using standard linear model methods with covariate correction. Function enrichment, eQTL/sQTL comparisons, and eQTL/sQTL-GWAS colocalization were used to characterize the molecular QTLs.

*Longissimus dorsi* muscle tissue was consistently collected from the 6th to 7th rib region of 582 F2 hybrid pigs derived from crosses between European White Duroc and Chinese Erhualian breeds (Figure 1). Whole-genome sequencing (WGS) of these individuals yielded an average of 214,301,970 cleaned paired-end reads per sample, corresponding to a mean sequencing depth of 12.68× (Supplementary Table S1). Alignment of these reads to the *Sus scrofa* reference genome (Sscrofa11.1) [26] achieved a high mapping rate exceeding 99% (Supplementary Table S1). Variant calling identified 44,003,932 polymorphic sites, including 35,739,686 single nucleotide polymorphisms (SNPs) and 8,264,246 small insertions and deletions (Indels, <50 bp), with 21,079,318 variants meeting the quality criteria for downstream analyses (Figure 1).

Total RNA was extracted from the same muscle tissue samples and sequenced using strand-specific paired-end RNA-seq libraries. On average, 83,051,308 reads per sample were aligned to the Sscrofa11.1 genome [26] using HISAT2 [31], with 96% of reads successfully aligning (Supplementary Table S2). After quality control and normalization, 13,707 genes and 66,208 intron clusters were identified. To assess the similarity of these expression profiles to bulk RNA profiles from seven major tissues (muscle, heart, adipose, brain, liver, small intestine, and blood) in the pigGTEx database [12], principal component analysis (PCA) was conducted. Our samples clustered with publicly available muscle tissue data, thereby confirming the reliability of our expression profiles for further analysis (Supplementary Figure S2).

### 3.2. Cis-Heritability of Gene Expression and Intron Excision Ratios

Gene expression and splice junctions were considered heritable if they exhibited heritability (*h*^2^) > 0 and met the significance threshold of *p* < 0.05. In total, 11,239 genes exhibited expression heritability attributable to cis-variants (*h*^2^ > 0, *p* < 0.05), with an average heritability of 0.20 ± 0.16 (mean ± standard deviation). Similarly, 10,773 intron excision ratios showed heritability explained by cis-variants (*h*^2^ > 0, *p* < 0.05), with a mean heritability of 0.18 ± 0.24 (Supplementary Figure S3).

### 3.3. Identification and Characterization of Muscle eQTL

**Figure 2:**
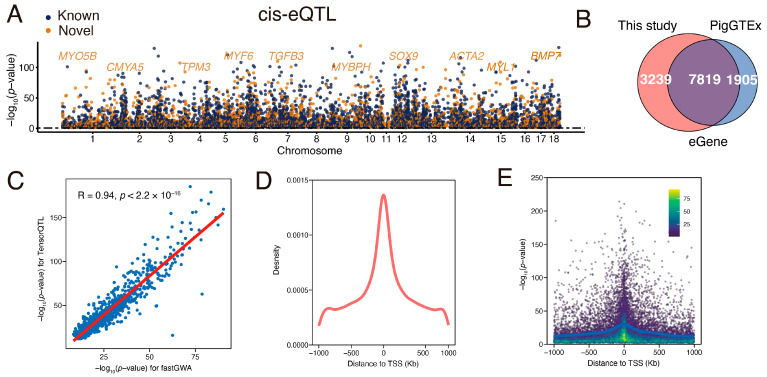
Characterization of cis-eQTLs. (**A**) Manhattan plot illustrating lead cis-eQTLs for each eGene, where blue and orange points denote previously known and newly identified eGenes, respectively. A subset of eGenes associated with muscle function is labeled for clarity. (**B**) Overlap of eGenes between this study (N = 582) and PigGTEx muscle studies (N = 1321). (**C**) The correlation of significance levels (−log_10_(*p*-value)) of cis-eQTLs (blue points) was estimated by linear mixed models (LMM) using fastGWA and linear models (LM) employing TensorQTL. The red line represents the linear regression line. (**D**) Density distribution of significant cis-eQTLs around TSS for each eGene. (**E**) Distribution of *p*-values for significant cis-eQTLs associated with each eGene.

To identify autosomal loci influencing gene expression, we performed cis-eQTL mapping using TensorQTL [39], adjusting for hidden confounders (PCs and PEER factors) as covariates. This analysis identified 11,058 genes (representing 80.67% of total expressed genes) with at least one significant cis-eQTL (FDR < 0.05), hereafter referred to as eGenes (Figure 2A). When compared with public muscle cis-eQTL datasets from pigGTEx (N = 1321) [12], the majority of eGenes (70.71%) identified in this study overlapped with previously reported eGenes, while more than 3239 eGenes (29.29%) were newly discovered (Figure 2B). These newly identified eGenes include key genes involved in muscle development and contraction, such as *TPM3* [46], *MYF6* [47], *SOX9* [48], and *MYL1* [49], providing an important complement to the current database (Figure 2A). To further validate our cis-eQTL results, we applied a linear mixed model (LMM) using fastGWA [38]. We observed a strong correlation (*R* = 0.94) between the significance levels of genetic variants estimated by LMM and those obtained using linear regression (implemented in TensorQTL) in a random subset of 1000 genes, supporting the robustness of our findings (Figure 2C).

The overall distribution of cis-eQTLs in this study is consistent with previous findings in humans, pigs, cattle, and chickens [9,10,11,12], with most muscle cis-eQTLs clustering near the TSS of target genes (Figure 2D). In total, 25.98% of significant cis-eQTLs were located within 100 kb of the TSS. We found an enrichment of low *p* values closer to TSSs, showing that cis-eQTLs are more likely to be located within this distance (Figure 2E). Additionally, we annotated cis-eQTLs with chromatin state predictions from the muscle tissue [50]. As expected, we observed that cis-eQTLs were most significantly enriched in TSS-proximal transcribed regions and enhancers (Supplementary Figure S4), showing these eQTLs fell in functionally relevant genomic regions involved in gene expression regulation.

### 3.4. Identification and Characterization of Muscle sQTL

**Figure 3:**
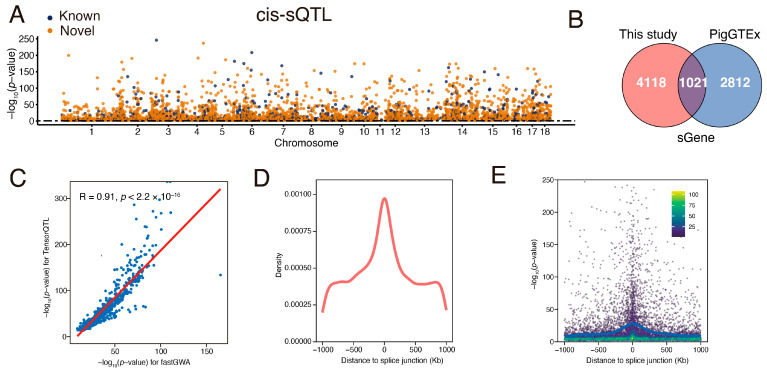
Characterization of cis-sQTLs. (**A**) Manhattan plot illustrating lead cis-sQTLs for each eGene, where blue and orange points denote previously known and newly identified eGenes, respectively. (**B**) Overlap of sGenes between this study (N = 582) and PigGTEx muscle studies (N = 1321). (**C**) The correlation of significance levels (−log_10_(*p*-value)) of cis-sQTLs (blue points) was estimated by linear mixed models (LMM) using fastGWA and linear models (LM) employing TensorQTL. The red line represents the linear regression line. (**D**) Density distribution of significant cis-sQTLs around the junctions for each sGene. (**E**) Distribution of *p*-values for significant cis-sQTLs associated with each sGene.

Effects of genetic variation on RNA splicing have been strongly implicated in complex economic traits [51,52]. To explore RNA splicing regulation in the porcine muscle, we identified local alternative splicing events by quantifying intron excision ratios using LeafCutter [35]. As a result, we identified 5139 significant sGene (FDR < 0.05) with at least one significant cis-sQTL, of which more than 4118 sGenes (80.13%) were newly discovered compared with the pigGTEx database [12] (Figure 3A,B). Correlation analysis further validated our cis-sQTL findings (Figure 3C).

Positional enrichment of cis-sQTL reveals a clustering pattern near splice junctions, with 29.81% of sQTLs located within 100 kb of these junctions, indicating that genetic variants close to splicing sites have a large effect (Figure 3D,E). Similar to cis-eQTL, cis-sQTLs were also strongly enriched in TSS-proximal transcribed regions and enhancers, albeit at relatively lower enrichment levels (Supplementary Figure S4).

### 3.5. Independent Regulation of eQTLs and sQTLs

**Figure 4:**
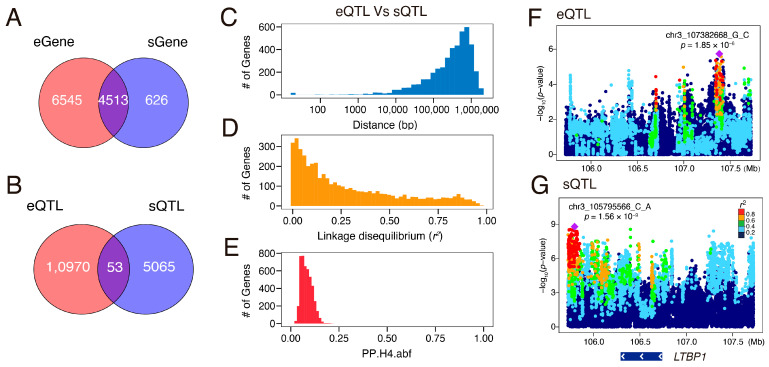
Comparative analysis of eQTLs and sQTLs. (**A**) Overlap of eGenes and sGenes. (**B**) Overlap of eQTLs and sQTLs. The distribution of distances (bp) (**C**), linkage disequilibrium (*r*^2^) (**D**), and posterior probabilities of colocalization (PP.H4.abf) (**E**) between lead variants of the same eGene and sGene. (**F**,**G**) Manhattan plots displaying association signals (−log_10_(*p*-values)) for eQTL (**F**) and sQTL (**G**) analyses of *LTBP1*. Each dot represents a genetic variant, colored according to LD with the lead variant.

We further analyzed the overlap between eGenes and sGenes, as well as between eQTLs and sQTLs. As a result, most of the genes associated with sQTLs (87.81%) were also identified as eGenes (Figure 4A). However, despite this overlap at the gene level, the lead variants were rarely shared between eQTLs and sQTLs, with only 53 instances of direct overlap observed (Figure 4B).

Further examination of the genomic distribution of lead variants for the same genes showed that these variants were typically located between 10 kilobases (kb) and 1 megabase (Mb) apart (Figure 4C). Moreover, these variants exhibited low linkage disequilibrium (LD), with an average *r*^2^ of 0.14 (Figure 4D), indicating largely independent regulatory mechanisms governing gene expression and alternative splicing.

Colocalization analysis further supported this notion, as the average posterior probability of shared causal variants (PPH4) was only 0.08 (Figure 4E), underscoring the limited convergence of regulatory control between eQTLs and sQTLs. A representative example is the *LTPB1* gene, where distinct genomic loci were responsible for regulating expression and splicing (Figure 4F,G). Notably, the LD between the respective lead variants was lower than 0.01, reinforcing the hypothesis that gene expression and splicing are predominantly governed by independent genetic architectures.

### 3.6. Colocalization Analysis of eQTL and sQTL with GWAS Locus

**Figure 5:**
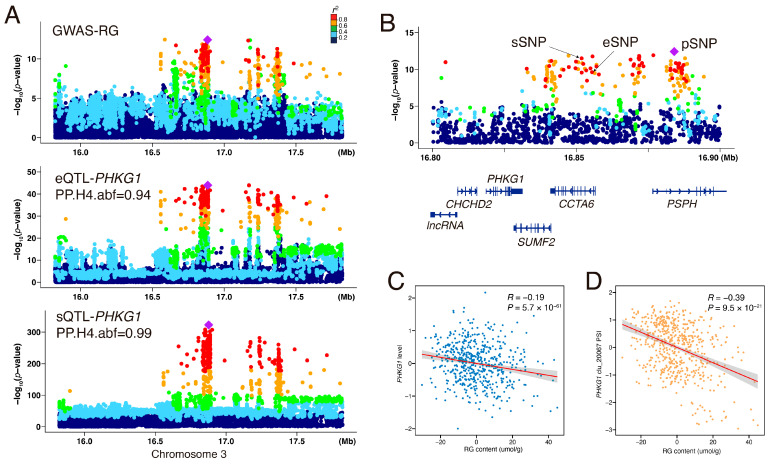
The eQTL and sQTL of *PHKG1* colocalized with GWAS signals of RG traits. (**A**) Manhattan plots of GWAS signals for residual glycogen (RG) content alongside eQTL and sQTL signals (3:16,830,087:16,830,325: clu_20067) for the *PHKG1* gene. The lead eQTL is indicated by a purple diamond. Each dot represents a variant, and its color represents the LD degree between the lead eQTL and rest ci-eQTL. (**B**) Detailed view of the lead SNPs for RG lead GWAS signal (pSNP), lead eQTL corresponding to *PHKG1* gene expression (eSNP), and lead sQTL linked to *PHKG1* intron excision ratios (sSNP). The position of these SNPs relative to the *PHKG1* gene is indicated, with adjacent gene annotations provided below. (**C**,**D**) The correlation between RG content and *PHKG1* gene expression (**C**), as well as the correlation between RG content and *PHKG1* intron excision ratios (**D**). Orange and red dots represent individual samples; the red line shows the linear regression fit, with the shaded grey area indicating the 95% confidence interval.

To investigate the role of muscle eQTLs and sQTLs in regulating meat traits in pigs, we performed a colocalization analysis of eQTLs and sQTLs with GWAS loci. First, we conducted a GWAS for total glycogen (TG) and residual glycogen (RG) content in the *longissimus dorsi* muscle using the same 546 F2 individuals. Consistent with our previous QTL findings based on the 60 K BeadChip [41], the GWAS identified the most significant locus on chromosome 3 (chr3) for both TG and RG (Supplementary Figure S5; Figure 5A).

Further colocalization analysis using coloc [43] revealed strong colocalization of *PHKG1* eQTL and sQTL signals with GWAS loci, with the sQTL showing a higher posterior probability (PP.H4 = 0.99) than the eQTL (PP.H4 = 0.94), suggesting that alternative splicing of *PHKG1* may play a more critical role in glycogen metabolism (Figure 5A). Additionally, we validated the colocalization of the *PHKG1* sQTL and GWAS signals in an independent F7 crossbred population, which also supported this association, albeit with a slightly lower posterior probability (PP.H4 = 0.87, Supplementary Figure S6). Linkage disequilibrium (LD) analysis further supported this finding, showing that the lead GWAS pSNP and corresponding sSNP/eSNP were in high LD (Figure 5B), indicating a shared regulatory mechanism. Moreover, correlation analysis demonstrated a significant negative relationship between *PHKG1* expression and RG content (*R* = −0.19, *p* = 5.7 × 10^−6^, Figure 5C), with an even stronger correlation observed for alternative splicing as measured by PSI scores (*R* = −0.39, *p* < 9.5 × 10^−21^, Figure 5D). These findings highlight the pivotal role of *PHKG1* splicing regulation in glycogen metabolism and suggest that sQTLs may be a more influential determinant of meat quality traits than eQTLs.

### 3.7. The Alternative Splicing of Exon 10 of the PHKG1 Affects Glycogen Metabolism

We further investigated the molecular mechanism through which *PHKG1* splicing modulates glycogen metabolism. Previous biochemical studies established *PHKG1*’s central role in glycogen phosphorylase activation and identified exon 10 dys-splicing as a modulator of enzymatic activity through targeted qPCR/RT-PCR and enzyme activity assay [41]. Our transcriptome-wide analysis now provides population-level evidence that alternative splicing of exon 10 constitutes the primary regulatory mechanism underlying observed sQTL effects.

**Figure 6:**
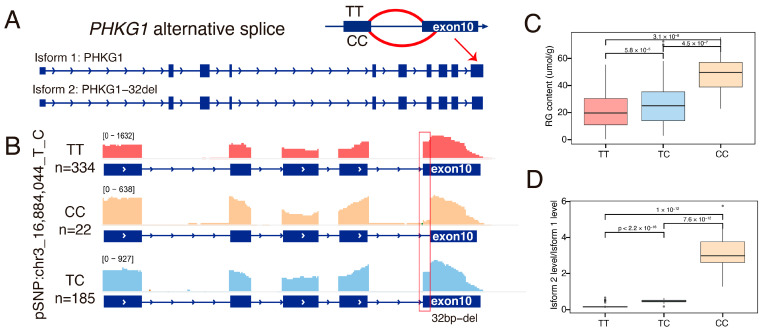
The exon10 splice event in the *PHKG1* gene and its association with RG traits. (**A**) Schematic representation of the *PHKG1* exon10 splicing event, producing two transcript isoforms: isoform 1 (PHKG1), which includes the full exon 10 sequence, while isoform 2 (PHKG1-32del), which carries a 32 bp deletion in exon 10. (**B**) The IGV visualization illustrates genotype-specific splicing patterns (TT, CC, and TC), displaying read distributions across exon10 junctions for different haplotypes of pSNP chr3:16,884,044_T_C. The red box highlights the read coverage over the 32-bp deletion region, indicating a lack of read support in this region for individuals with the CC haplotype due to the deletion. (**C**,**D**) Boxplot showing the difference in RG content (**C**) and the expression ratios of isform2 and isform1 (**D**) explained by the haplotype (TT, CC, and TC) of pSNP chr3_16,884,044_T_C, excluding 41 individuals with missing genotypes (./.). *p*-values were calculated by two-sided Student’s *t*-test.

Utilizing splice junction analysis with LeafCutter [35], we identified two competing splice acceptor sites within exon 10 that produce two distinct transcript isoforms (Figure 6A). In TT genotype individuals (reference allele), preferential use of the proximal splice site, results in full-length transcripts (isoform 1) that retain the complete exon 10 sequence (Figure 6B). Conversely, the CC genotype (mutant allele) promotes the utilization of a distal splice site, creating a frameshifted isoform2 with 32-bp deletion of exon 10 (Figure 6B, Supplementary Figure S3). This truncated transcript has been shown to induce nonsense-mediated decay (NMD), leading to lower protein levels and impairing glycogen breakdown [41]. Consistent with previous findings, mutant CC individuals exhibited a significantly higher proportion of truncated transcripts, resulting in impaired glycogen metabolism and consequently higher residual glycogen (RG) content (Figure 6C,D). Collectively, our findings underscore the role of *PHKG1* alternative splicing in glycogen metabolism and highlight the critical contribution of sQTL analysis to identity casual gene for complex traits.

### 3.8. The PHKG1 Splice Site Usage Is Significantly Associated with GP-Related Meat Quality Traits

Building on the evidence that *PHKG1* alternative splicing modulates glycogen metabolism, we further evaluated its impact on additional meat quality parameters in the F_2_ population. Specifically, we investigated the association between splice site usage—quantified by percent spliced-in (PSI) values—and key phenotypes linked to glycolytic potential (GP), including ultimate pH, drip loss, intramuscular fat (IMF) content, and muscle color.

As previously shown, preferential use of the proximal splice site (chr3:16,830,087:16,830,325; clu_20067), was consistently associated with lower residual and total glycogen levels. Importantly, this splicing pattern also showed positive associations with downstream meat quality traits, such as a moderate increase in postmortem pH (LDpH24h: *p* = 1.03 × 10^−2^, *T* = 2.57) and higher Minolta L (LDL24h: *p* = 4.56 × 10^−2^, *T* = −2), indicative of a more favorable meat quality profile (Table 1). Conversely, increased usage of the distal splice site (chr3:16,830,087:16,830,357), resulting in a 32-nt deletion and truncated *PHKG1* transcripts, was associated not only with elevated glycogen content but also reduced pH and meat color (Table 1). This splicing pattern also exhibited trends toward increased drip loss and reduced IMF content—two traits closely tied to meat palatability and water-holding capacity—though these associations did not reach statistical significance (Table 1).

Overall, these results underscore the broader phenotypic consequences of *PHKG1* splice site usage, highlighting its regulatory influence not only on muscle energy metabolism but also on postmortem meat quality. Preferential splicing toward the proximal site is associated with a suite of traits indicative of favorable meat quality, while distal site usage aligns with less desirable outcomes.

## 4. Discussion

This study presents a comprehensive atlas of eQTLs and sQTLs in porcine *longissimus dorsi* muscle, offering novel insights into the genetic regulation of transcriptomic variation underlying meat quality traits. Our analysis of 582 F2 crossbred pigs significantly expands the catalog of regulatory loci in pigs, identifying 29% of eGenes and 80% of sGenes as previously unannotated in the PigGTEx database [12]. This highlights the utility of high-resolution RNA-seq from uniform muscle tissues coupled with large-scale genomic data in uncovering tissue region-specific regulatory mechanisms, particularly for splicing events, which have historically been underestimated in farm animals [53].

The distinct genomic distributions of eQTLs and sQTLs underscore their divergent regulatory roles. The enrichment of eQTLs near transcription start sites (TSS), particularly in regions marked by active TSS-proximal transcribed regions and enhancers, is consistent with their proposed role in modulating transcriptional initiation and expression [50]. In contrast, the preferential localization of sQTLs near splice junctions suggests their direct involvement in spliceosome recruitment or exon recognition [54]—This is consistent with observed in both human studies and agriculturally relevant animals [9,10,11,12]. Moreover, despite a substantial overlap at the gene level (87.81% of sGenes are also eGenes), the genetic regulation of expression and splicing appears to operate largely independently. Only a few lead variants overlap between eQTLs and sQTLs, with lead variants for co-regulated genes typically separated by distances of 10 kb–1 Mb and exhibiting minimal linkage disequilibrium (average *r*^2^ = 0.14). Colocalization analysis further supports this independence, showing a low posterior probability of shared causal variants for the same e/sGenes (average PPH4 = 0.08). These findings imply that genetic variants influencing transcription and splicing are predominantly governed by independent genetic architectures, necessitating complementary analytical approaches to fully dissect their contributions to complex traits.

A key application of our atlas lies in the integration of e/sQTL mapping with GWAS signals for muscle glycogen-related traits (total glycogen [TG] and residual glycogen [RG]). Colocalization analysis revealed strong evidence (PP.H4 = 0.99 for sQTL vs. 0.94 for eQTL) that alternative splicing of *PHKG1* exon 10, rather than its overall expression, drives phenotypic variation in glycogen metabolism. In mutant (CC) individuals, preferential utilization of a cryptic splice acceptor site results in a 32-bp deletion in exon 10. This splice shift has been reported to generate a truncated transcript subject to nonsense-mediated decay, reducing *PHKG1* protein levels and impairing glycogen breakdown [41]. This is consistent with the observed negative correlation between exon 10 retention (PSI) and RG content (*R* = −0.39, *p* = 9.5 × 10^−21^). While prior studies implicated *PHKG1* in porcine glycogen metabolism, our results directly link its splicing regulation to meat quality variation, demonstrating that sQTLs can pinpoint causal mechanisms obscured by eQTL-focused analyses. This underscores the critical role of post-transcriptional regulation in bridging genetic associations to functional outcomes.

The disproportionately high number of novel sQTLs (~80%) identified in this study, compared to novel eQTLs (~29%), underscores the greater regulatory complexity and tissue-specificity of splicing in porcine muscle. Several factors likely contribute to this observation. First, skeletal muscle is anatomically heterogeneous, comprising over 47 distinct regions [16], each with unique transcriptional programs [17,18]. Our standardized sampling from the 6th–7th rib region minimized spatial variability and may have improved the resolution of splicing signals compared to broader datasets like PigGTEx. A similar effect was reported by Mapel et al., where consistent sampling in testis increased sGene detection more than 4 times (7000 vs. 1573) compared with cattleGTEx despite only a twofold increase in sample size (117 individuals vs. 60 individuals) [55]. Second, strand-specific RNA sequencing enabled more accurate detection of splice junctions, particularly for antisense transcripts [56]. While direct evidence linking strand specificity to increased sQTL discovery is limited, its advantage in identifying cis-natural antisense transcripts (cis-NATs)—as shown by Mukherjee et al., who reported 918 novel NATs—suggests potential indirect gains in splicing resolution [57]. Third, splicing regulation is inherently more complex and variable than gene expression. Comparative studies report only ~2% conservation of alternative splicing loci between humans and mice, despite 86% exon conservation [58]. Additionally, sQTLs often involve distal or intronic regulatory elements [9,12] and show higher tissue specificity than eQTLs—as seen in chickenGTEx, where 32.1% of sQTLs were tissue-specific versus 10.6% for eQTLs [11]. Together, the high proportion of novel sQTLs identified in porcine muscle likely reflects both the biological complexity of splicing regulation and the benefits of standardized sampling and strand-specific sequencing. These findings underscore the value of precise anatomical sampling and high-resolution transcriptomics for mapping isoform-level regulation and advancing our understanding of complex traits in livestock.

Our atlas provides an expanded source of regulatory variants in the porcine genome. Several limitations and challenges of this study warrant consideration. First, our focus on an F2 cross-population captures limited genetic diversity, with extended LD blocks reducing fine-mapping resolution. Second, the functional interpretation of non-coding variants requires integration with epigenomic datasets (e.g., ATAC-seq, methylation, or histone modification profiles) to pinpoint causal regulatory elements. Third, bulk tissue analysis obscures cell type-specific regulatory effects addressable through muscle single-cell RNA-seq in future.

## 5. Conclusions

In conclusion, this study elucidates the genetic landscape of gene expression and splicing regulation in porcine *longissimus dorsi* muscle, revealing distinct mechanisms underlying eQTLs and sQTLs. By integrating eQTL/sQTL with GWAS loci, we confirmed the alternative splicing of *PHKG1* as a pivotal modulator of glycogen metabolism. These findings underscore the utility of integrative transcriptomic approaches in pinpointing causal genes for complex economic traits, which will contribute to the advancement of genetic improvement programs in future porcine breeding.

## Figures and Tables

**Table 1:** Effect of *PHKG1* splice site usage on meat quality traits of *longissimus dorsi* muscle in F2 population.

Abbreviation	Traits	*R* ^2^	*p*-Value	*T*-Value
Proximal splice site: chr3:16,830,087:16,830,325: clu_20067				
LDRG	Residual Glycogen, mmol/g	0.15	9.59 × 10^−21^	−9.73
LDTG	Total Glycogen, mmol/g	0.08	1.57 × 10^−11^	−6.89
LDpH24h	pH 24 h of LM	0.01	1.03 × 10^−2^	2.57
LDL24h	Minolta L value 24 h of LM	0.01	4.56 × 10^−2^	−2.00
LDEZ24h	24-h drip loss of LM, %	0.00	1.98 × 10^−1^	−1.29
LDintFAT	Intramuscular fat content of LM	0.00	6.65 × 10^−1^	0.43
Distal splice site: chr3:16,830,087:16,830,357: clu_20067 (32-del)				
LDRG	Residual Glycogen, mmol/g	0.15	4.74 × 10^−21^	9.82
LDTG	Total Glycogen, mmol/g	0.08	1.85 × 10^−11^	6.86
LDpH24h	pH 24 h of LD	0.01	1.25 × 10^−2^	−2.51
LDL24h	Minolta L value 24 h of LD	0.01	5.78 × 10^−2^	1.90
LDEZ24h	24-h drip loss of LD, %	0.00	2.08 × 10^−1^	1.26
LDintFAT	Intramuscular fat content of LD	0.00	6.68 × 10^−1^	−0.40

## Data Availability

The eQTL and sQTL results can be accessed on GitHub at https://github.com/zhoudreames/Pig_eQTL-sQTL/ (accessed on 21 April 2025).

## Supplementary Materials

The following supporting information can be downloaded at: https://www.mdpi.com/article/10.3390/ani15091209/s1, Supplementary Figures S1–S6 and Tables S1–S3 can be found in the supplementary files. Figure S1. The posterior variance of the PEER factor weights for gene expression and splicing values. Figure S2. Clustering analysis of F2 muscle samples and seven tissues from pigGTEx based on the normalized gene expression. Figure S3: Distribution of cis-heritability for gene expression and intron excision ratio. Figure S4: Enrichment analysis of cis-eQTLs and cis-sQTLs with 15 chromatin states in the muscle. Figure S5: Manhattan plots showing genome-wide association for total glycogen (TG) and Residual glycogen (RG). Figure S6 Validation of the *PHKG1* sQTL signal in an independent F7 crossbred pig population. Table S1: Summary of WGS samples for the F2 population. Table S2. Summary of RNA-seq samples for the F2 population. Table S3: Summary of genotype-specific splicing patterns for RG phenotype.

## Abbreviations

The following abbreviations are used in this manuscript:

eQTL	Expression quantitative trait loci
sQTL	Splice quantitative trait loci
GWAS	Genome-wide associated studies
RG	Residual glycogen content
TG	Total glycogen content
WGS	Whole-genome sequencing
TSS	Transcription start site
PCA	Principal component analysis
LD	Linkage disequilibrium
FDR	False discovery rate
