# Supplementary material for: Identification of Gene Expression and Splicing QTLs in Porcine Muscle Associated with Meat Quality Traits
Source: Animals (Basel). 2025 Apr 24;15(9):1209. doi: 10.3390/ani15091209 (PMC12071002; doi:10.3390/ani15091209)
Supplement: Supplementary file 1 [file animals-15-01209-s001.zip › Supplementary_Figure.pdf]

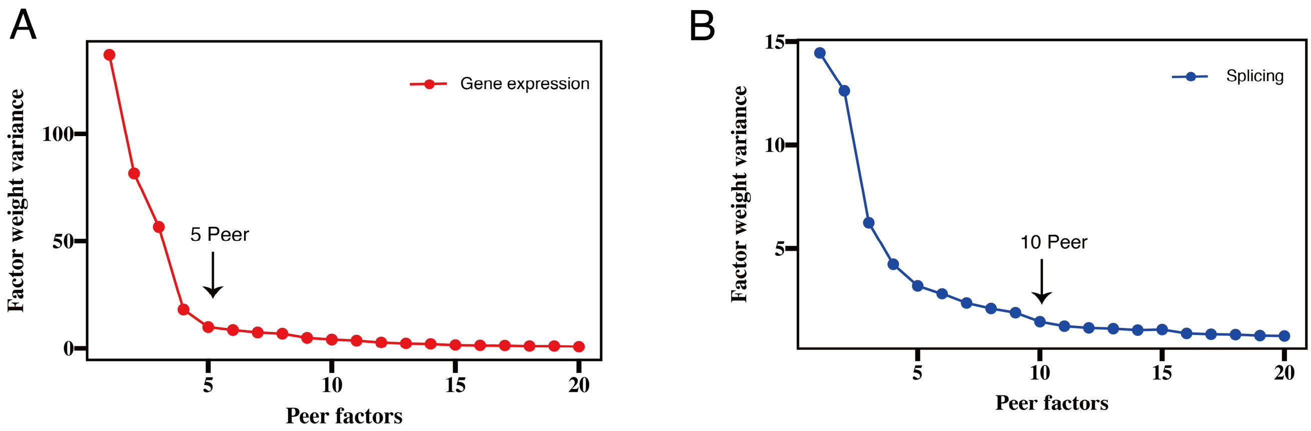

Figure S1. Posterior variance of the PEER factor weights for gene expression and splicing values.

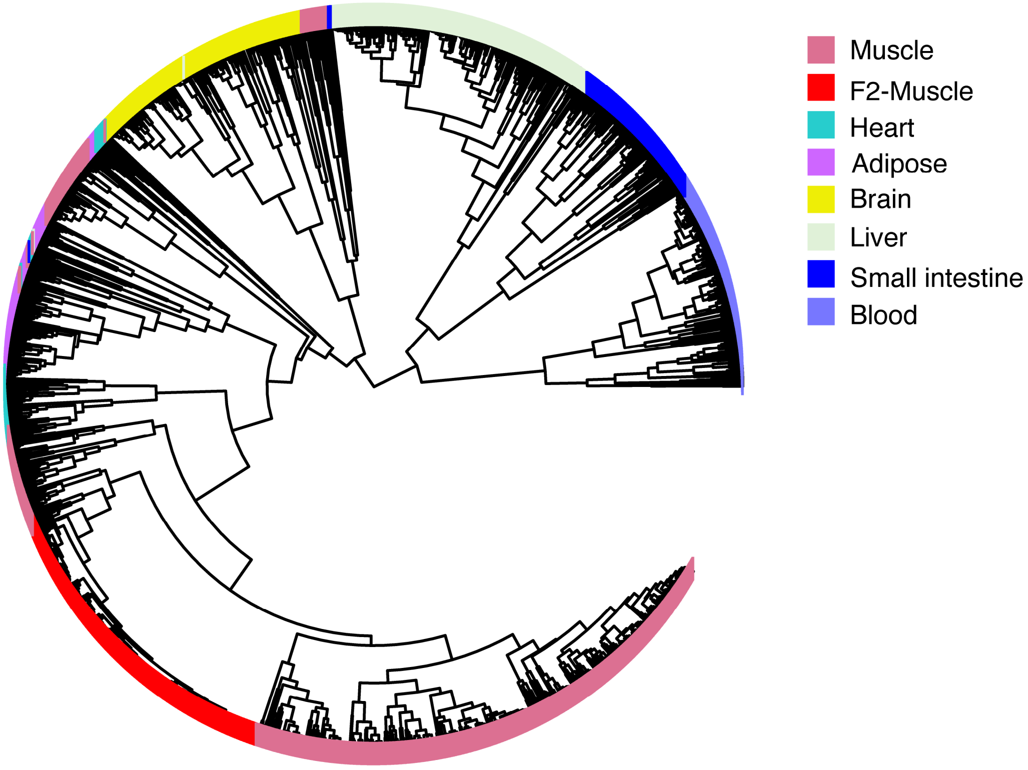

Figure S2. Clustering of F2 muscle samples and seven tissues from pigGTEx based on the normalized expression (log10 -transformed TPM) of 5,000 highly variable genes, defined as the top 20% of genes with the largest s.d.

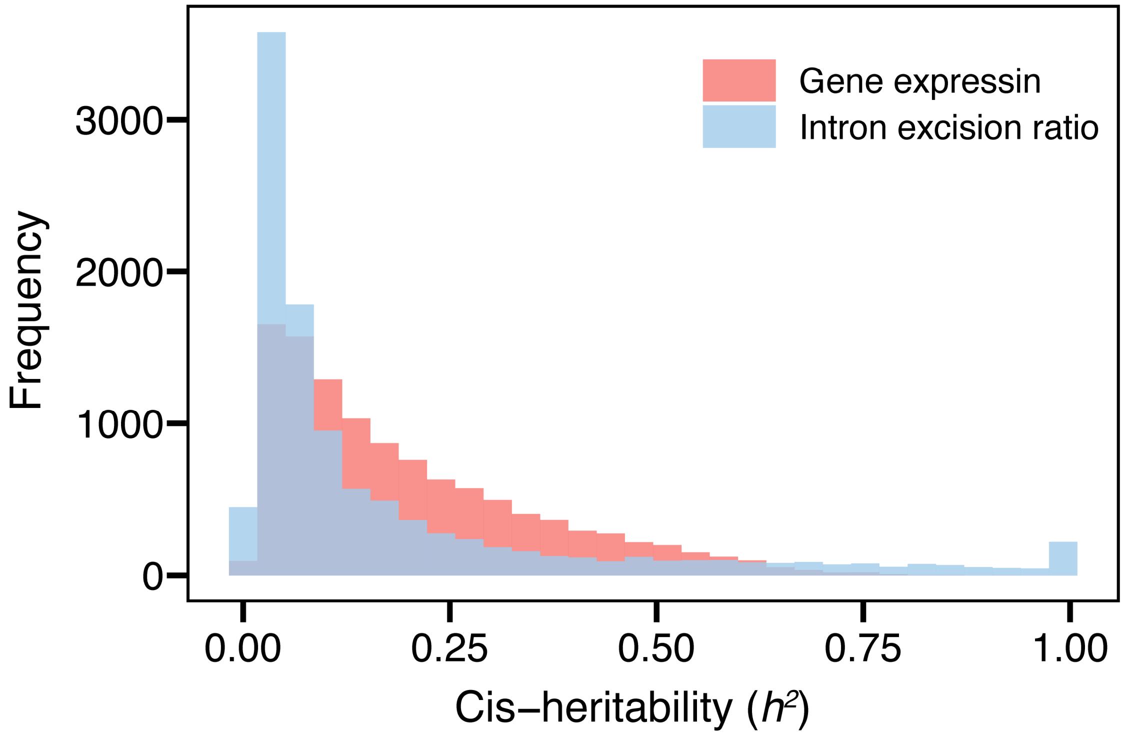

Figure S3. Heritability of 11,239 genes expression and 10,773 intron excision ratio explained by cis-SNPs

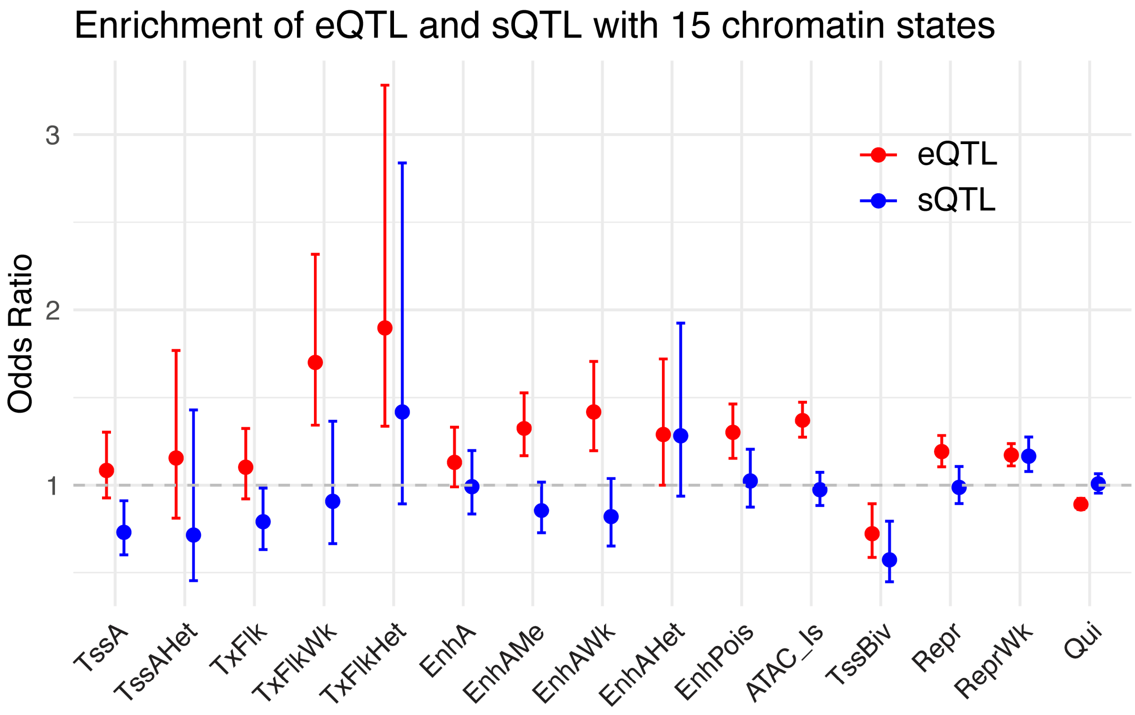

Figure S4. Enrichment anlaysis of cis-eQTLs and cis-sQTLs with 15 chromatin states in the muscle, respectively. The point and error bars indicate the odds ratio and 95% CI.

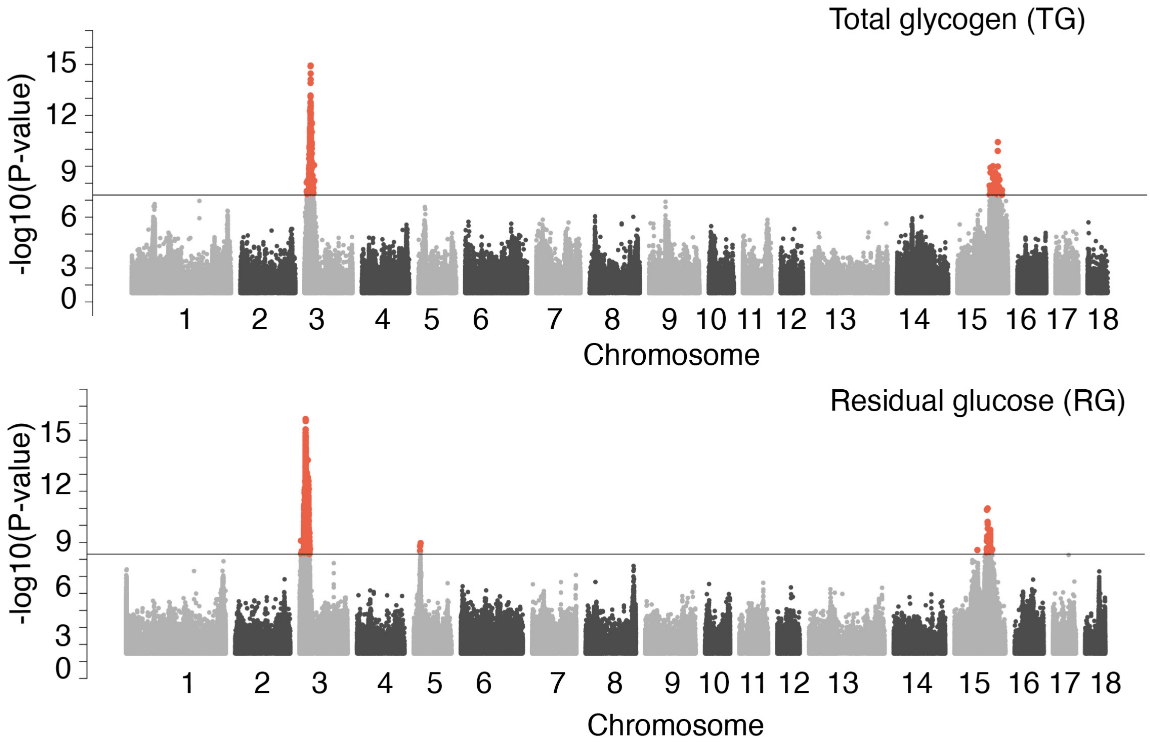

Figure S5 Manhattan plots showing genome-wide association for (A) total glycogen (TG) and (B) Residual glucose (RG). Variants that are associated with trait at a significance threshold of $p < 5e{-8}$ are highlighted in red.

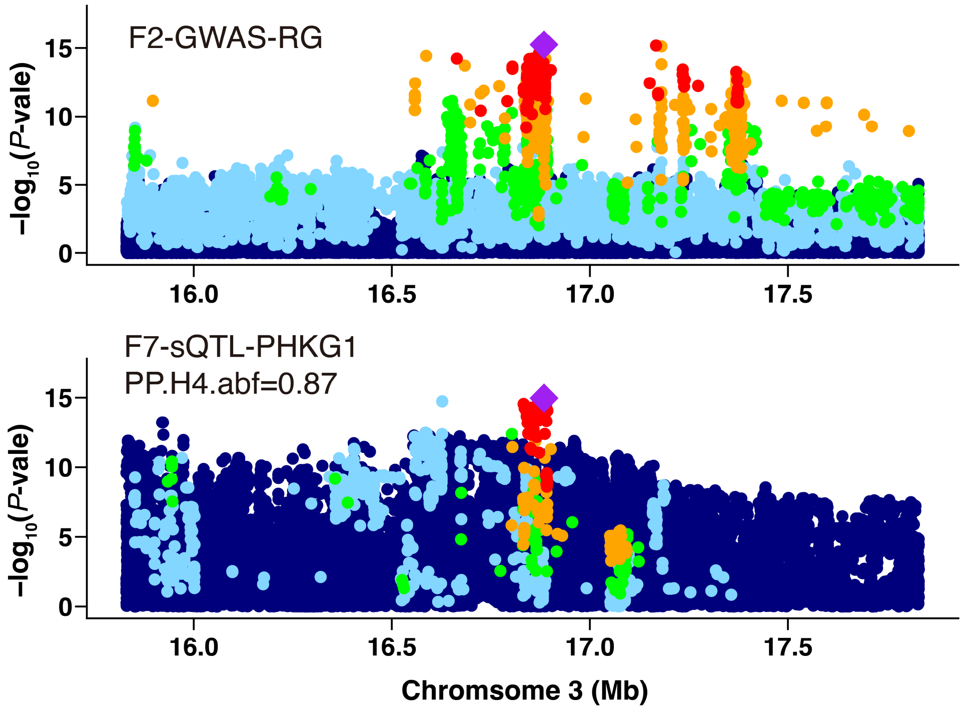

Figure S6 Validation of the PHKG1 sQTL signal in an independent F7 crossbred pig population. Manhattan plots showing the GWAS signals for residual glycogen (RG) content in the F2 population alongside the sQTL signals for the PHKG1 gene in the F7 crossbred pigs.
